# Hypopigmenting Effects of Brown Algae-Derived Phytochemicals: A Review on Molecular Mechanisms

**DOI:** 10.3390/md15100297

**Published:** 2017-09-24

**Authors:** Mohammed Shariful Azam, Jinkyung Choi, Min-Sup Lee, Hyeung-Rak Kim

**Affiliations:** 1 Department of Food Science and Nutrition, Pukyong National University, 45 Yongso-Ro, Nam-Gu, Busan 48513, Korea; azam_dof@yahoo.com (M.S.A.); kamadky@gmail.com (M.-S.L.); 2 Department of Foodservice Management, Woosong University, Daejeon 34606, Korea; choi3728@wsu.ac.kr

**Keywords:** brown algae, hyperpigmentation, meroterpenoid, molecular mechanism, phlorotannins

## Abstract

There is a rapid increase in the demand for natural hypopigmenting agents from marine sources for cosmeceutical and pharmaceutical applications. Currently, marine macroalgae are considered as a safe and effective source of diverse bioactive compounds. Many research groups are exploring marine macroalgae to discover and characterize novel compounds for cosmeceutical, nutraceutical, and pharmaceutical applications. Many types of bioactive secondary metabolites from marine algae, including phlorotannins, sulfated polysaccharides, carotenoids, and meroterpenoids, have already been documented for their potential applications in the pharmaceutical industry. Among these metabolites, phlorotannins from brown algae have been widely screened for their pharmaceutical and hypopigmenting effects. Unfortunately, the majority of these articles did not have detailed investigations on molecular targets, which is critical to fulfilling the criteria for their cosmeceutical and pharmaceutical use. Very recently, a few meroterpenoids have been discovered from *Sargassum* sp., with the examination of their anti-melanogenic properties and mechanisms. Despite the scarcity of in vivo and clinical investigations of molecular mechanistic events of marine algae-derived hypopigmenting agents, identifying the therapeutic targets and their validation in humans has been a major challenge for future studies. In this review, we focused on available data representing molecular mechanisms underlying hypopigmenting properties of potential marine brown alga-derived compounds.

## 1. Introduction

Skin pigmentation is a complex process that includes synthesis of melanin pigments in melanocytes at the basal layer of epidermis and their distribution to neighboring keratinocytes. Skin color is primarily determined by the type, intensity and distribution of melanin pigments. The role of melanin is also essential to protect the skin from deleterious effects of solar ultraviolet (UV) radiation, which is responsible for the excess generation of reactive oxygen species (ROS) and leads to photocarcinogenesis [1], premature skin aging [2] and other damages. Although melanin pigments have an important role in protecting the skin against photocarcinogenesis, irregular hyperpigmentation is not desirable and is considered as a skin pigmentary disorder. Hyperpigmentation is caused by abnormal accumulation of melanin pigments in the skin with major skin hyperpigmentation disorders including melasma, freckles, lentigo, moles, leukoplakia, and so on [3,4]. Furthermore, periorbital hyperpigmentation, post-inflammatory hyperpigmentation, drugs and cosmetics-induced hyperpigmentation, age spots and hormonal factor-mediated hyperpigmentation are also common factors in causing imbalanced pigmentation in the skin [5,6]. There are various commercial depigmenting agents currently being used in skin-whitening cosmetics. However, most of them are not completely satisfactory due to their low efficiency or safety concerns [6,7,8]. Hydroquinone, which was introduced for clinical use as a depigmenting agent since 1961, has been widely used for years to treat skin hyperpigmentation disorders, especially against melasma [6,9]. However, it has been reported to cause contact dermatitis and exogenous ochronosis, which is darkening of the area of skin exposed to hydroquinone [9]. It was also reported to be unstable in cosmetics and is an easily-oxidized ingredient in cosmetic formulations [9]. Considering the consequences of long-term treatments [10], its application in cosmetics has been banned since 2001 by the European Commission [6]. Arbutin, a terrestrial plant originated derivative of hydroquinone, is another commercial skin-whitening agent. Although it showed strong tyrosinase (TYR) inhibitory activity, it was shown to lack efficiency in melanocyte pigmentation and in clinical trials [8]. As it is a glycosylated hydroquinone, arbutin may have similar risks as hydroquinone [7,11]. Kojic acid is an effective skin-lightening agent, but previous studies reported that kojic acid is associated with allergic contact dermatitis [12] and enhancement of carcinogenicity [13]. Therefore, there is growing demand for the examination of effective and safe agents from natural sources to treat hyperpigmentation disorders and to use in skin-whitening cosmetics in Asian countries.

More than 70% of the Earth’s surface consists of a marine habitat, which is a huge source of diversified population of flora and fauna [14]. Among marine biota, marine algae are rich in a diverse range of beneficial compounds with strong safety profiles [15,16,17]. Marine macroalgae are broadly divided into three major groups: brown algae (phaeophyceae); red algae (rhodophyceae), and; green algae (chlorophyceae). Brown algae are specifically rich sources of phenolic compounds with high antioxidant activities [18] and, thus, it could be a useful ingredient for nutricosmetics [19]. Marine phenolic compounds, such as phlorotannins, only exist in brown algae and possess a high potential for pharmaceutical and nutraceutical applications [18,20]. Brown algae are also rich in meroterpenoids that are proven for diverse health benefits due to their strong antioxidant activities [21,22,23,24,25,26,27]. Marine algae-derived sulfated polysaccharides, such as fucoidan, laminarans, ulvan and carrageenan, are being utilized in food, cosmetic and pharmaceutical industries [15]. It is important to note that cosmeceuticals from natural sources have higher market value compared to synthetic compounds [20]. There are many studies examining the hypopigmentation and skin protection roles of flavonoids, most of which are of terrestrial origin [28]. Until now, a considerable amount of effort has been given on searching hypopigmenting compounds from terrestrial sources. However, there are limited data on the hypopigmenting activities of marine alga-derived compounds focusing on their molecular mechanistic aspects.

Understanding the molecular mechanisms of any bioactive compound is very important for their proper application. A lack of clear understanding on therapeutic targets and effects results in unfortunate outcomes with huge economic losses during drug discovery. Therefore, after the initial screening, it is very important to know the detailed molecular events and identify specific targets of potential compounds in specific cell types and suitable animal models before entering the pre-clinical and clinical trials in the long process of drug development. In this review, we will summarize the potential hypopigmenting role of marine brown alga-derived compounds focusing on their molecular mechanistic approach. We will also focus on current status, major gaps and future directions on this issue.

## 2. Major Pathways Involved in Melanogenesis

**Figure 1:**
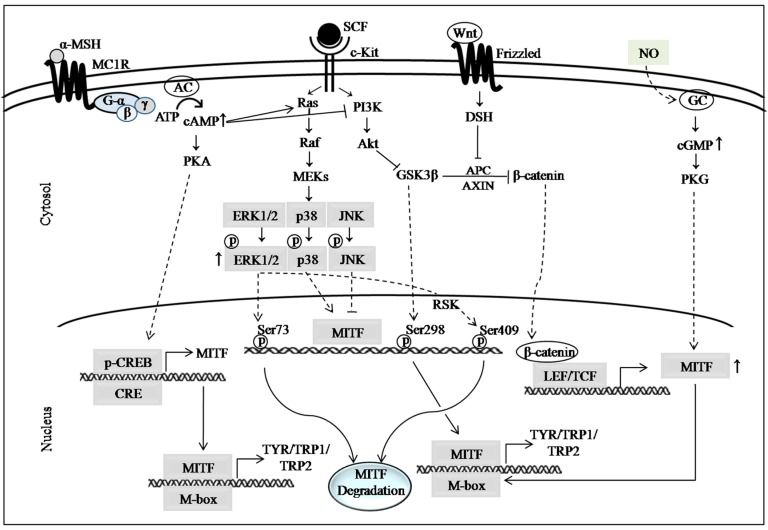
Schematic diagram representing major signaling pathways involved in the expression of melanogenic enzymes [29,30,31,32,33,34].

There are several well documented signaling pathways that are involved in the regulation of skin pigmentation. Among them, the cyclic adenosine monophosphate (cAMP) signaling pathway is considered as the classical signaling pathway that plays a pivotal role in melanogenesis [29]. Other major signaling pathways are also recognized for mediating melanogenic pathways, including mitogen-activated protein kinase (MAPK) signaling, phosphatidylinositol 3-kinase (PI3K)/Akt signaling and Wnt/β-catenin signaling pathways (Figure 1). The cAMP signaling pathway also initiates cross-talk with the extracellular signal-regulated kinase (ERK) 1/2, PI3K/Akt and Wnt/β-catenin signaling pathways. The molecular events in these pathways are briefly described in the following sections.

### 2.1. cAMP Signaling Pathway

The cAMP signaling pathway initiates when extracellular signals, such as α-melanocyte stimulating hormone (α-MSH) or adrenocorticotrophic hormone (ACTH), binds to the melanocortin 1 receptor (MC1R), which is a G-protein coupled seven transmembrane receptor. The conversion of cellular ATP to cAMP, the second messenger in melanogenic process, is catalyzed by adenylate cyclase (AC) once it is activated by G-protein. There are several stimuli, such as UV radiation, α-MSH, ACTH, forskolin, isobutylmethylxanthine or other cAMP elevating agents, which stimulate cellular responses to cAMP induction. Elevated levels of cAMP activate protein kinase A (PKA) through binding of cAMP to the binding domain of PKA. PKA consists of regulatory subunits and catalytic subunits. In the unstimulated state, the two catalytic subunits of PKA remain bound to the regulatory subunit dimer as an inactive holoenzyme, which has two cAMP-binding domains on each regulatory subunit [35]. Binding of cAMP to regulatory subunits of PKA results in the dissociation and activation of its catalytic subunits, which leads to the translocation of PKA to the nucleus and phosphorylation of its substrate cAMP-response element-binding protein (CREB) [5,36]. There are two reported phosphorylation residues in CREB, such as Ser129 and Ser133, which are associated with its activation. Ser133 is considered as the major site of phosphorylation for its activation [37]. Nonetheless, Jeong et al. [38] demonstrated the anti-melanogenic effects of baicalin through inhibition of CREB phosphorylation at Ser129, while phosphorylation level at Ser133 residue remained unchanged. CREB is one of the major transcription factors for the expression (or activation) of microphthalmia-associated transcription factor (MITF), which is responsible for the regulation of melanogenic enzymes [39]. Once CREB is activated, it binds to the CRE motif of the MITF promoter and transactivate this key regulator of melanogenic enzymes: TYR, tyrosinase-related protein (TRP)1, and TRP2 (Figure 1) [40,41]. Although these three enzymes are involved in melanin synthesis, the TYR is considered as the rate limiting enzyme for melanin synthesis [42]. In the cAMP signaling pathway, there are several critical points where skin-whitening compounds are involved in exerting their hypopigmenting effects. The major therapeutic target or regulatory points are: suppression of cAMP induction [30], inhibition of cAMP binding to PKA [36,43], inhibition of CREB activation and MITF downregulation [30], and inhibition of MC1R expression [33].

### 2.2. MAP Kinase Signaling Pathways

MAPK signaling is initiated by the activation of the c-Kit receptor, which is also known as tyrosine kinase receptor, with stem cell factor (SCF). The paracrine factor SCF is primarily produced by keratinocytes in response to several stimuli. After stimulation with SCF, the c-Kit transduces the signal that culminates in posttranslational modification of MITF, which alters its transcriptional activity or stability [32]. This signal can be mediated by three different kinases, such as extracellular signal regulated kinase (ERK), c-Jun N-terminal kinase (JNK) and p38 MAPK. Among them, ERK1/2 signaling is the most widely studied and strong signal, which is also considered a negative feedback mechanism for melanogenesis [44]. ERK1/2 becomes activated upon phosphorylation by Raf/MEK, before becoming translocated to the nucleus where they phosphorylate MITF at the Ser73 residue. This leads to subsequent ubiquitin-dependent proteasomal degradation of MITF [45,46]. Furthermore, they phosphorylate MITF at Ser409 through ribosomal S6 kinase (RSK), which also results in proteasome-mediated degradation of MITF [47]. ERK1/2-mediated degradation of MITF is recognized as one of the most attractive therapeutic target for anti-melanogenic compounds [48]. However, a previous study reported that ERK1/2 activation did not result in phosphorylation of MITF at the Ser73 residue, although they confirmed degradation of MITF [49]. Another study demonstrated that proteasomal degradation of MITF is not always followed by ERK activation [38]. ERK1/2 signaling also has cross-talk with cAMP signaling as the elevation of cellular cAMP can activate Ras/ERK signaling to induce MITF degradation [41,50].

The phosphorylation and activation of p38 MAPK in B16 melanoma cells was found to increase melanin synthesis through increased expression of MITF and TYR via CREB phosphorylation [51,52]. A previous investigation using an anti-melanogenic compound, c-phycocyanin, from spirulina, showed suppressed TYR expression through inhibition of p38 MAPK-mediated CREB activation [46]. Similarly, another study also reported the positive correlation between the suppression of p38 MAPK phosphorylation and the downregulation of MITF [33]. However, they did not clarify whether p38 directly acts on MITF phosphorylation or via CREB in this study. Phosphorylation and activation of stress-activated protein kinase, JNK, has also been reported to inhibit melanogenesis via downregulation of MITF [53]. On the other hand, Han et al. [54] demonstrated the positive correlation of suppression of total JNK with downregulation of MITF and TRP1 leading to attenuated melanogenesis and TYR activity in B16F10 cells. However, JNK-mediated suppression of pigmentation by marine algae-derived compounds has not yet been reported by any study.

### 2.3. PI3K/Akt Signaling Pathway

Similar to MAPK signaling, PI3K/Akt signaling cascade is also initiated by SCF/c-Kit interaction. Involvement of PI3K/Akt signaling in the melanogenic process was reported by several studies [55,56]. Akt, also known as protein kinase B, is a serine/threonine protein kinase. It is a downstream signaling molecule to PI3K. Akt is activated by phosphorylation on Ser473 and Thr308 residues via activation of PI3K [57]. After being activated, Akt phosphorylates the Ser9 residue of glycogen synthase kinase 3β (GSK3β), resulting in it being transformed into its inactive form [58]. However, a previous study in B16 cells reported that cAMP inhibits Akt phosphorylation and activation, which leads to activation of GSK3β by its dephosphorylation [29]. The activated form of GSK3β phosphorylates MITF at the Ser298 residue, which enhances the binding of MITF to the M-box sequence of the TYR promoter [29]. This cross-talk of cAMP with PI3K/Akt signaling cascade was reported in a previous study to occur via inhibition of PI3K activity in B16F10 cells [59]. Therefore, the phosphorylation and activation of PI3K/Akt could be a potential therapeutic approach for skin hyperpigmentation disorders. The Akt signaling pathway also has cross-talk with the Wnt/β-catenin signaling, which works via the Akt/GSK3β/β-catenin cascade [60].

### 2.4. Wnt/β-Catenin Signaling Pathway

The Wnt/β-catenin signaling pathway has been reported by several studies, which is involved in melanin synthesis [60,61]. There are several Wnt ligands that activate Wnt/β-catenin signaling, such as Wnt1, Wnt3a and Wnt8. Wnt-1 has been known to enhance melanocyte expansion and differentiation in mouse neural crest cells [62]. Activation of the frizzled receptor and low-density lipoprotein receptor-related protein 5/6 co-receptor by Wnt ligands phosphorylate and inactivate GSK3β, which results in increased cytoplasmic level of β-catenin. The accumulated β-catenin translocates into the nucleus and acts as a coactivator of MITF in association with lymphocyte enhancer factor 1 (LEF1), resulting in the transactivation of MITF. In the absence of Wnt signaling, a protein complex consisting of axin, adenomatous polyposis coli (APC), GSK3β and casein kinase 1 (CK1) leads to the phosphorylation of β-catenin in the N-terminal serine/threonine residues, which is followed by its proteasomal degradation. A previous study demonstrated that andrographolide, a plant-originated diterpenoid, inhibited melanin synthesis through decreased phosphorylation of GSK3β and enhanced degradation of β-catenin in B16F10 cells, human epidermis melanocytes (HEM), and ultraviolet B (UVB)-induced guinea-pig skin [60]. Suppression of pigmentation in normal human melanocytes (NHM) was caused by the enhancement of proteasome-dependent β-catenin degradation by cardamonin [61]. A previous study also reported cross-talk between cAMP signaling and Wnt/β-catenin signaling pathways in melanoma cells and NHM [63]. In this study, they showed α-MSH stimulated the activation of PKA and attenuated GSK3β. They also demonstrated that cAMP/PKA-mediated phosphorylation of β-catenin at Ser675 residue led to its stability and transcriptional activity. Therefore, the inhibition of GSK3β phosphorylation or enhancement of β-catenin degradation could be a potential therapeutic target for hypopigmenting compounds against skin hyperpigmentation disorders. However, the anti-melanogenic activity of marine macroalga-derived compounds via Wnt/β-catenin signaling has not yet been reported.

### 2.5. Autophagy, Nitric Oxide (NO) Signaling and Other Mechanistic Targets of Hypopigmenting Agents

Autophagy is a process through which damaged or defective organelles and unnecessary cellular aggregates are degraded by autophagosome-lysosomal machinery, which is known to facilitate the recycling of nutrients during starvation. Similarly, autophagy has a role in the destruction and removal of defective melanosomes [64]. Only a few investigations have reported the regulation of melanogenesis by the activation of autophagy in melanocytes, although the role of autophagy in melanogenic process is not well established [64,65]. Kim et al. [65] demonstrated ARP101(C_20_H_26_N_2_O_5_S)-induced activation of autophagy in α-MSH-stimulated Melan-a cells by punctate structure formation with autophagy marker green fluorescence protein fused-LC3 protein (GFP-LC3), and increased conversion of LC3I to LC3 II protein. Moreover, by electron microscopic analysis, they demonstrated the engulfment of melanosomes by autophagosomes, which resulted in the downregulation of pigmentation. In another study, Van Den Boorn et al. [66] reported suppression of pigmentation by monobenzone-induced polyubiquitination of TYR and macroautophagocytic degradation of melanosomes in human melanoma cells.

The NO signaling pathway was also reported to regulate melanogenesis [67]. Keratinocytes produce NO in response to UV-stimulation and NO interacts with guanylate cyclase (GC), which leads to increased levels of cyclic guanosine monophosphate (cGMP) in melanocytes. An elevated level of cGMP induces melanogenesis through the enhancement of MITF expression. UV-induced NO was also shown to enhance melanogenesis in alpaca skin melanocytes by stimulating MITF phosphorylation, while MITF levels remained unchanged [68]. Moreover, NO stimulated keratinocytes to produce excess α-MSH and also induced cAMP signaling by elevated expression of MC1R in melanocytes [69]. The inhibition of melanosome maturation [70] and trafficking to surrounding keratinocytes [71] were also reported to inhibit melanogenesis. However, these signaling mechanisms have not yet been reported in the case of marine macroalga-derived compounds.

## 3. Hypopigmenting Effects of Marine Brown Algae-Derived Phenolic Compounds

**Figure 2:**
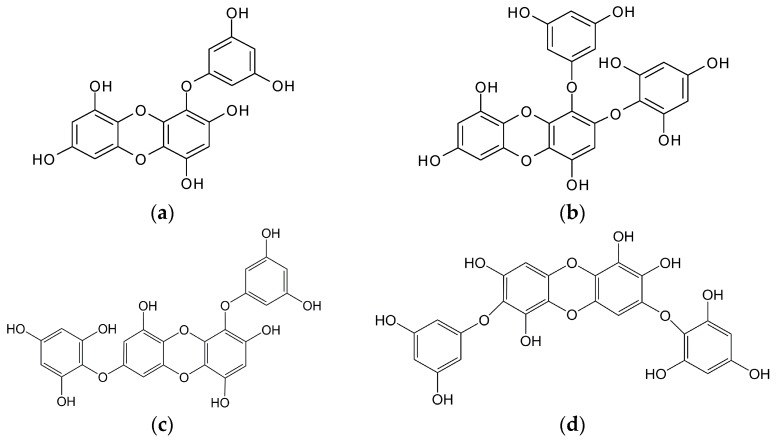

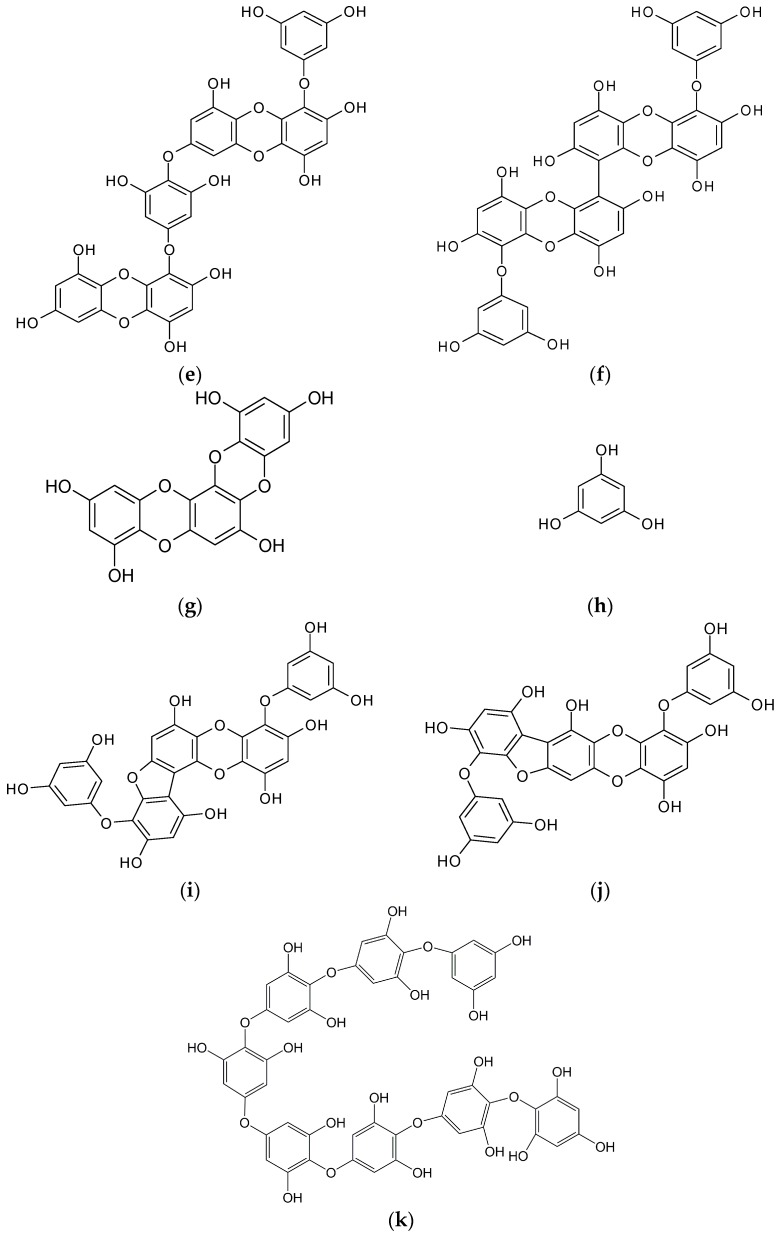
Chemical structure of phlorotannins isolated from brown algae: (**a**) Eckol; (**b**) 2-phloroeckol; (**c**) 7-phloroeckol; (**d**) Diphlorethohydroxycarmalol; (**e**) Dieckol; (**f**) 6,6′-Bieckol; (**g**) Dioxinodehydroeckol; (**h**) Phloroglucinol; (**i**) Phlorofucofuroeckol A; (**j**) Phlorofucofuroeckol B; and (**k**) Octaphlorethol A.

Phlorotannins, polymers of phloroglucinol (1,3,5-trihydroxybenzene), are marine polyphenols found only in brown algae (Figure 2 and Table 1) [20]. These compounds, also known as phloroglucinol derivatives, are markedly different from polyphenols of terrestrial origin. Algal phlorotannins have a wide range of applications, including anti-melanogenic [18,55,72], anti-skin aging [73], anti-oxidant [18,74,75], anti-inflammatory [76], hepatoprotective [74] and anti-allergic [77] activity. It is noteworthy that most of these properties of phlorotannins are valuable in the development of nutraceuticals and cosmeceuticals. Among brown alga-derived phlorotannins, phloroglucinol [78], eckol [78], dieckol [18,78], diphlorethohydroxycarmalol [79] and octaphlorethol A [72,80] were reported as potent TYR inhibitors, although there are a lack of in vitro and in vivo study on detailed molecular mechanisms of hypopigmenting action of these compounds. Heo et al. [18] isolated three types of phlorotannins, such as phloroglucinol, eckol and dieckol, from *Ecklonia cava*, and demonstrated the anti-melanogenic activity of eckol and dieckol on the basis of inhibition of mushroom TYR and melanin synthesis in B16F10 cells. In this study, they identified dieckol as a promising skin-whitening agent. Kang et al. [78] screened seventeen marine algae using mushroom TYR and identified phloroglucinol, eckstolonol, eckol, dieckol and phlorofucofuroeckol A from *E. stolonifera* as TYR inhibitors. They also reported dieckol as a potent TYR inhibitor (IC_50_ 2.16 μg/mL), which showed activity three times higher than that of kojic acid. Our research team studied the hypopigmenting properties of another phlorotannin, dioxinodehydroeckol (isolated from *E. stolonifera*), in α-MSH-stimulated B16F10 cells and found PI3K/Akt-mediated transcriptional downregulation of MITF, leading to downregulation of melanogenic enzymes, which are namely TYR, TRP1 and TRP2 [55]. Cha et al. [7] screened aqueous extracts of 43 types of marine algae based on mushroom TYR inhibition and identified a red alga, *Schizymenia dubyi* (IC_50_ 9.08 μg/mL), and three brown algae, *Endarachne binghamiae* (IC_50_ 27.16 μg/mL)*, Sargassum siliquastrum* (IC_50_ 19.85 μg/mL) and *E. cava* (IC_50_ 18.00 μg/mL) as potent TYR inhibitors. They further demonstrated the inhibitory effects of *E. cava* and *S. siliquastrum* on TYR activity and melanin synthesis in both B16F10 cells and Zebrafish model. Interestingly, in their investigations, the extracts of *E. cava* caused strong TYR inhibition (92%) in B16 cells, although it was much weaker (48%) in Zebrafish. However, they did not report any molecular event in this study. Jang et al. [81] isolated 4-hydroxyphenethyl alcohol from a brown alga, *Hizikia fusiformis.* They demonstrated inhibition of mushroom TYR activity and melanin content in B16F10 cells and remarkable reduction of UVB-induced hyperpigmented spots in brown guinea-pig skin after eight weeks of topical application. They also did not report any molecular mechanisms in hypopigmentation in their study.

## 4. Hypopigmenting Effects of Meroterpenoids

**Figure 3:**
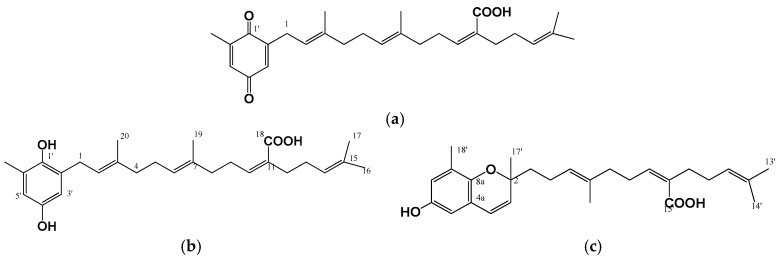
Chemical structure of anti-melanogenic meroterpenoids isolated from the brown alga, *Sargassum serratifolium* [30]: (**a**) Sargaquinoic acid; (**b**) Sargahydroquinoic acid; and (**c**) Sargachromanol.

The brown algae are rich sources of meroterpenoid compounds, which are beneficial to general health [21,30,87]. In particular, the *Sargassum* genus was reported to contain high amount of meroterpenoids [21]. Algal meroterpenoids have anti-inflammatory [21,87,88,89,90], antioxidant [22], anti-ageing [23], anti-atherosclerotic [24,91], anti-adipogenic [25,92], anti-diabetic [26], anti-carcinogenic [93,94] and neuroprotective [95] activities. Recently, we demonstrated the hypopigmenting effects of ethanolic extract from *S. serratifolium* in B16F10 cells and identified three active meroterpenoid compounds, including sargahydroquinoic acid, sargaquinoic acid and sargachromenol (Figure 3), on the basis of their inhibitory activity on melanin synthesis in α-MSH-stimulated B16F10 cells [30]. We also elucidated that the extract from *S. serratifolium* inhibited hyperpigmentation in B16F10 cells through regulation of MITF via cAMP/CREB and ERK signaling pathways (Table 1). To the best of our knowledge, there was no examination of the anti-melanogenic activity of algal meroterpenoids before this report.

## 5. Hypopigmenting Effects of Fucoxanthin

Fucoxanthin is a group of carotenoids found in brown algae. The information on the effects of fucoxanthin on melanogenesis is very limited. Fucoxantin was reported to suppress TYR activity and melanogenesis in B16 murine melanoma cells. Furthermore, this has been seen in vivo in guinea pig and mouse skin [85]. In mice, the suppression of melanin biosynthesis was reported by both topical and oral treatments with fucoxanthin, although topical treatments resulted in better effects. This study has provided an important focus on the expression levels of melanogenic receptors in UV-irradiated mice and guinea pig skin. They found that topical treatment of 1% fucoxanthin significantly suppressed mRNA levels of endothelin receptor A (EDNRA), p75 neurotrophin receptor (p75NTR), prostaglandin E receptor 1 (EP1) and MC1R in mice. It also suppressed COX-2 expression, which downregulates prostaglandin (PG) in epidermis. Interestingly, although fucoxantin slightly suppressed TYR mRNA expression, there was no significant suppression. Therefore, they reported that fucoxanthin mainly suppressed TRP1 instead of the TYR. They suggested the suppression of PG and its receptor, EP1, in addition to MC1R by fucoxanthin, which has an inhibitory effect on melanogenesis. They also demonstrated the suppression of pigmentation in guinea pigs by a daily intake of low amount of fucoxanthin (0.001% in diet). Therefore, it can be a promising candidate for the formulation of cosmeceutical.

## 6. Hypopigmenting Effects of Non-Phenolic Compounds

Fucoidans, a fucose-rich sulfated polysaccharide, are predominantly found in marine brown algae and echinoderms [96]. Fucoidans have been shown to inhibit the activity of TYR [84,97], matrix metalloproteases (MMPs) and elastase [98]. Several studies indicated the high potential of fucoidan for anti-skin aging [96], anti-inflammatory [99] and antioxidant [100] activity, which demonstrates its cosmetic potential. Several previous articles reviewing bioactivity and therapeutic potential of fucoidans had a distinct lack of information regarding its hypopigmenting activity [101,102]. However, in a clinical test with 20 subjects, Fitton et al. [96] demonstrated that brown alga, *F. vesiculosus* extract (containing 58.6% fucoidan and 33.7% polyphenol) at 0.3% (*w*/*v*) attenuated the melanin index of age spots, increased brightness and reduced wrinkles. In another study, fucoidan, isolated from *F. vesiculosus,* was demonstrated to attenuate melanin content in spontaneously immortalized murine melanocyte Mel-Ab cells via ERK-mediated downregulation of MITF and TYR expression [84]. In contrast, a recent study had no inhibitory effects of fucoidan, isolated from the same alga, on TYR, TRP1, TRP2 and MITF expression in B16 murine melanoma cells, although it inhibited cellular melanin and TYR activity [42]. They suggested that fucoidan upregulated TYR expression, which was positively correlated to its apoptosis-stimulating activity in melanoma cells. Moreover, their data indicated that fucoidan suppressed the biological activity of TYR, which leads to inhibition of melanin synthesis. However, there is still very limited information on the depigmenting effects of fucoidans. Therefore, extensive studies will be needed regarding the screening of hypopigmenting fucoidans and characterization of their mechanistic events in vitro and in vivo.

## 7. Current Gaps and Future Directions

The necessity for identification and utilization of novel pharmaceuticals, cosmeceuticals and nutraceuticals from marine bioresources has been realized by many researchers. The world market for hypopigmentation is also huge for these marine-derived bioactive products. However, the sustainable use of marine resources, standardization of isolation process ensuring bioavailability and efficiency, safety profiling of isolated compounds and optimization of suitable doses are important. The stability of active compounds is also important for their commercial utilization. The development of an aquaculture technique for target algae could be a good option to ensure a sustainable supply for commercial utilization. The harvesting season and maturation stage are also important as there are seasonal and age variation in the biochemical composition and amount of some active components in marine algae.

The bioactivity of many compounds depends on their chemical structure and conformation, bioavailability, as well as type and position of functional groups. There are several novel extraction and separation techniques, such as supercritical CO_2_ extraction, membrane separation and ultrasonic-aided extraction. Depending on the properties of target compounds, the proper extraction and separation techniques should be selected to overcome the variances of source material. In some cases, the proper screening technique is also important for finding out the hypopigmentation compounds or extracts. In many cases, the activities of anti-melanogenic compounds are frequently verified against the inhibitory activity of mushroom TYR. However, this is not completely suitable, as there are inherent differences between mushroom and human TYR [103,104]. If human TYR is not readily available, the cellular TYR from mouse melanocytes can be much more reliable than mushroom TYR for initial screening of anti-melanogenic agents [86]. The phylogenetic analysis of TYR from various organisms on the basis of amino acid sequence of central catalytic domains suggests high level of homologies among human, gorilla and mouse TYR (representing the same cluster), while mushroom TYR was considerably more heterologous and placed in a distant cluster [103]. A previous study reported no effects of ethanolic extract of *S. polycystum* and its hexane fraction on mushroom TYR, but results in significant inhibition of cellular TYR when being tested on B16F10 mouse melanoma cells [86]. Han et al. [54] also reported that hydrolyzed ginseng extract could not inhibit mushroom TYR, but markedly suppressed melanogenesis and TYR activity in B16F10 cells. Similar findings were also reported by other studies [105]. Therefore, instead of the mushroom TYR inhibition assay, the human or mouse TYR inhibition assay could be a useful tool for screening potential anti-melanogenic agents.

In addition to screening techniques for hypopigmentation compounds, the safety of potential hypopigmenting compounds is essential for the application of human cosmetics or cosmeceuticals. Some commercial skin-whitening compounds, such as hydroquinone, have been used to treat skin hyperpigmentation disorders for a long time and were finally banned for their unwanted side effects. Therefore, long-term pre-clinical and clinical trials are necessary to verify the activity in humans focusing on molecular events, efficiency and side effects.

## 8. Conclusions

Anti-melanogenic compounds from marine brown algae have high potential as therapeutic agents against skin hyperpigmentation disorders. They also have huge demand in cosmetic industries as skin-lightening agents due to the increasing concern for lighter skin complexion by Asian women [106]. In association with efficiency, the safety profiles of hypopigmentation compounds are very important for human use. Marine algae are considered as being diversified and safe sources of bioactive compounds. Therefore, the identification of novel hypopigmentation compounds from marine algae and uncovering their molecular mechanisms using in vitro and in vivo systems has great importance in meeting the future challenges of pharmaceutical and cosmetic industry.

## Figures and Tables

**Table 1:** Overview of major hypopigmenting compounds from marine brown algae.

Algae	Compounds/Extract	Type	Action Mechanism	Experimental System	Reference
*Ecklonia stolonifera*	Dioxinodehydroeckol	Phlorotannin	PI3K/Akt-mediated downregulation of MITF	B16F10 mouse melanoma cells	[55]
*Ishige okamurae*	Diphlorethohydroxycarmalol	Phlorotannin	Inhibition of mushroom TYR and melanin synthesis	B16F10 cells	[79]
*E. cava*	Eckol	Phlorotannin	Inhibition of cell free TYR (non-competitive) & cellular TYR, TRP1, and TRP2	B16F10 cells	[82]
	Dieckol		Inhibition of mushroom TYR & cellular melanin	B16F10 cells	[18]
	Dioxinodehydroeckol		Mushroom TYR inhibition	Cell free	[83]
	7-phloroeckol		Inhibition of mushroom TYR (non-competitive) & cellular melanin	B16F10 cells	[83]
*I. foliacea*	Octaphlorethol A	Phlorotannin	ERK1/2-mediated downregulation of MITF, TYR, TRP1 & TRP2 in B16. Inhibition of in vivo TYR activity and melanin synthesis	B16F10 cells, Zebra fish embryo	[72,80]
*Hizikia fusiformis*	4-hydroxyphenethyl alcohol	Non-flavonoid phenolic compound	Inhibition of mushroom TYR and melanin synthesis in B16. Reduction of pigmented spots in guinea-pig skin	B16F10 cells, Brown guinea-pig	[81]
*Fucus vesiculosus*	Fucoidan	Fucose-rich sulfated polysaccharide	ERK-mediated downregulation of MITF.	Mel-Ab cells	[84]
			Inhibition of cellular TYR activity, melanin content & cell proliferation	B16 murine melanoma cells	[42]
*Laminaria Japonica*	Fucoxanthin	Carotenoid	Reduced TYR activity in B16 and melanin content in guinea-pigs & mice skin. Suppress PGE2, MSH, TRP1 & melanogenic stimulant receptors, NTR, EP1 & MC1R in vivo	B16 murine melanoma, UVB-induced mice, & guinea-pig	[85]
*Sargassum serratifolium*	Ethanolic extract containing sargaquinoic acid, sargahydroquinoic acid & sargachromenol	Meroterpenoid	cAMP and ERK1/2-mediated downregulation of MITF	B16F10 cells	[30]
*S. polycystum*	Ethanolic extract & its hexane fraction	NR	Inhibition of cellular TYR & melanin production	B16F10 cells	[86]

EP1 = prostaglandin E receptor 1; MC1R = melanocortin 1 receptor; MSH = melanocyte stimulating hormone; NR = not reported; NTR = p75 neurotrophin receptor; PGE2 = prostaglandin E2; TYR = tyrosinase; TRP = tyrosinase-related protein; MITF = microphthalmia-associated transcription factor; ERK = extracellular signal-regulated kinase; cAMP = cyclic adenosine monophosphate.
